# Supplementary material for: Are carbon emissions trading and green financial instruments synergistic? -Comprehensive quantitative research based on content analysis
Source: PLoS One. 2024 Mar 7;19(3):e0298601. doi: 10.1371/journal.pone.0298601 (PMC10919601; doi:10.1371/journal.pone.0298601)
Supplement: S1 Data — (ZIP) [file pone.0298601.s001.zip › Data/地级市Z_F工作报告.zip!/║╙─╧/σo╤⌠╩╨/└┤╫╘╣1⁄2╓┌║┼í╛┬φ┐╦╩2╛▌═°í┐_2008.docx]

各位代表：

现在，我代表市人民政府，向大会作政府工作报告，请予审议，并请各位政协委员提出意见。

一、2007年工作回顾

2007年，市人民政府在省委、省政府和市委的正确领导下，高举中国特色社会主义伟大旗帜，深入贯彻落实科学发展观，团结带领全市人民开拓进取、扎实工作，全面完成了市五届人大四次会议确定的各项任务，实现了经济社会又好又快发展。初步统计，全年生产总值完成532.8亿元，比上年增长14.6%，分别比全国、全省平均增幅高3.2个和0.2个百分点；其中第一产业增长5.6%，第二产业增长17.1%，第三产业增长13.3%。人均生产总值达到1.5万元。地方财政一般预算收入完成23.2亿元，增长24.7%。全年粮食总产量达到241.9万吨，增长7.1%，再创历史新高。全部工业增加值完成311.2亿元，增长18.7%，其中规模以上工业增加值完成263.3亿元，增长20.5%；规模以上工业实现利润102.2亿元，增长21.2%。全社会固定资产投资完成252.3亿元，增长37.4%。金融机构各项存款余额达到364亿元，较年初增加22.7亿元，增长6.6%。各项贷款余额达到176.3亿元。城镇居民人均可支配收入达到11042元，增长23.2%，农民人均纯收入达到3460元，增长19%，分别高于全省平均增幅12.2个和6.8个百分点。全市人民共享了发展成果，真正得到了实惠。

2007年，我们突出抓了以下八个方面的工作：

（一）积极贯彻国家宏观调控政策，加快产业结构调整，转变经济发展方式。工业方面，按照走新型工业化道路的要求和“龙头带动、产业支撑、优化结构、集群发展”的思路，坚持“抓大带小”的工作方法，以骨干企业为依托，以重大项目建设为载体，以产业集群为平台，加大政策扶持、改革创新、资金投入和战略重组力度，企业的自主创新能力、资源利用率、精深加工比重、综合经济效益以及节能减排水平进一步提高，工业经济呈现强劲发展的势头。八大优势产业完成工业增加值250.1亿元，增长19.6%，占全市规模以上工业增加值的95%。全市“双超”企业达到133家，其中6户进入省百强企业行列。中原大化、濮耐分别入选全省百户重点企业和50户高成长性企业行列。龙宇化工、龙丰纸业等地方重点骨干企业发展势头良好。“桃园建民”等12件商标荣膺“河南省著名商标”。122个重点工业项目完成投资89.5亿元，为年计划的120.4%，有109个项目竣工投产。农业农村方面，全面落实各项支农惠农政策，加快农业结构调整，培植农业产业化龙头企业，农业综合生产能力进一步提高。新增有效灌溉面积2.2万亩、旱涝保收面积2.1万亩、节水灌溉面积8万亩，荣获全省“红旗渠精神杯”市级杯八连冠。全市优质专用小麦、优质水稻、优质玉米分别达到212万亩、70万亩和100万亩。建成无公害农产品、畜产品产地73个，无公害农产品、畜产品品种23个。全年肉、蛋、奶产量分别增长15.3%、11.9%和40.5%。畜牧业产值达到67.2亿元，较上年增加13.1亿元。发展林下经济7.2万亩，实现产值5.7亿元。农业产业化龙头企业达到420家，其中年产值超亿元的13家，带动农户39.8万户。全市综合机械化水平达到66%。全年转移劳动力60.5万人次，实现劳务收入45亿元。按照“示范带动、组织推动、产业强村、项目富民”和“抓两头、带中间”等要求和做法，我市社会主义新农村建设取得了新的经验和成效，被《中国新农村建设内参》专期介绍，成为中西部地区落实科学发展观，建设社会主义新农村的先进地区。第三产业方面，大力发展商贸流通业，全年社会消费品零售总额达到135.5亿元，增长17.8%。旅游业健康发展，全年接待游客245.2万人次，增长5.7%；旅游收入达到3.2亿元，增长6.7%。文化事业和文化产业发展迈出新步伐。濮阳县被评为“河南十大文化强县”，华晨杂技公司被评为“全国优秀出口文化企业”。大型现代戏《村官李天成》荣获全国第十届“五个一工程”入选作品奖。全市120个杂技演出团体在国内外巡回演出10000余场。房地产开发完成投资17.2亿元，增长24.4%。积极发展金融、信息、物流等现代服务业，全面提高第三产业占国民经济的比重，消费对经济增长的贡献率达到19%。

（二）狠抓县域经济和非公有制经济，筑牢发展基石。完善扶持政策，引导各县（区）立足比较优势，强化产业支撑、招商引资的理念，大力发展特色经济、民营经济、劳务经济，培育特色产业、大型企业集团，建设重大工业项目。同时，强化位次意识，以发展成效论政绩。2007年县（区）生产总值平均增幅在15%以上，形成了你追我赶的良好态势。适时出台了《关于鼓励支持和引导非公有制经济发展的实施意见》等政策措施，进一步加快了非公有制经济健康快速发展。止2007年年底，规模以上非公有制企业达到504家，其中年营业收入超千万元的436家，超亿元的75家。全市非公有制经济完成增加值277.1亿元，增长31.3%。非公有制经济占全市经济总量的52%，比上年提高3个百分点。

（三）狠抓关系全局和惠及民生的重大项目建设，切实增强濮阳发展后劲。根据国家加强宏观调控和调整产业结构的政策要求，我们抢抓国家重点支持农业、交通、能源、节能减排等基础产业和基础设施建设的机遇，结合濮阳实际，谋划实施了一批打基础、管长远的重大项目，增强了发展后劲。年初确定的186个重点项目，完成投资116.5亿元，有150个项目竣工投产。一批关系濮阳发展全局和惠及民生的重大项目建设取得突破。国电濮阳热电公司2×20万千瓦发电机组脱硫、各县污水处理厂、垃圾处理场等项目建成投用。濮水河综合治理工程基本完成。中原大化50万吨甲醇项目基本竣工。龙丰纸业25万吨轻量涂布纸、龙宇化工20万吨甲醇扩建等项目正在加快建设。黄河标准化堤防二期工程开工建设。永煤集团濮阳盐化工项目奠基。大广高速豫冀省界至南乐段已通过国土资源部土地预审。德商高速范县段工可研已通过评审。国电濮阳热电公司2×60万千瓦发电机组扩建工程预可研已报省发改委。长治至兖州复线铁路濮阳段正在开展前期工作。年初向全市人民承诺的实事基本完成。

（四）狠抓改革开放，不断为濮阳发展注入动力和活力。坚持以改革促发展。国有企业改革进一步深化，盘活存量资产16亿元，安置职工2.1万人，引进社会增量资金13亿元。通过引进永煤集团、省投资集团、香港嘉联公司、立马集团等战略投资者，对市甲醇厂、市氯碱厂、市三强热电、市三强纺织和气纺、市啤酒厂等企业成功实现了兼并重组和收购。与永煤集团签订了建设濮阳煤、盐化工园区战略合作协议。在现有年产20万吨甲醇项目扩建基础上，再新建年产20万吨和2×30万吨两个甲醇项目，使甲醇产能、装置规模达到年产100万吨；合作建设年产20万吨烧碱、25万吨聚氯乙烯、20万吨二甲醚、10万吨L—乳酸等项目。此外，还要和一些大企业、大集团合作开发建设一批化工、食品、林纸林板等关系濮阳长远发展的重大项目。这些重大项目建成后，濮阳GDP总量将超过千亿元，实现翻番。同时，积极推进文化体制、种子管理体制、水管体制、农村公路管养体制、粮食流通、统计、人事、城市信用社等领域的改革，均取得了阶段性成果。在资本市场发展上有了新突破，濮耐近期有望上市，蔚林化工、恒润化工等企业正在做上市前期工作。坚持以开放促发展。成功组织了“环渤海经济圈”招商活动，共签约项目30个，总投资32.2亿元。参加了中原文化港澳行暨2007豫港投资贸易洽谈会、第二届中博会、中原文化天津行等活动，举办了2007＇濮阳中华龙文化节暨杂技艺术节，共签约项目137个，总投资194.4亿元。实际利用外资6016万美元，增长30%。实际利用市外资金53.7亿元，增长33.1%。累计完成进出口总额2.6亿美元，其中外贸出口完成2.4亿美元。累计完成新签对外承包工程和劳务合作合同额 4.2亿美元，完成营业额4.9亿美元，外派人数达7305人，外经三项指标连续9年居全省首位。

（五）狠抓节能减排，切实推动资源节约型、环境友好型社会建设。加强重点企业、重点行业节能管理，加快淘汰落后生产能力，全年关停小火电机组11台，能力8万千瓦。以解除高新区“流域限批”为契机，深入开展环境综合整治，关闭拆除“十五小土”企业63家，关停污染严重的企业32家。同时，对群众反映强烈的金堤河水质严重污染问题进行了重点治理。深入推进土地三项整治和“禁实”工作，拆除实心黏土砖瓦窑厂326座，整理复垦土地6350亩。坚持节约集约用地，盘活存量土地2570亩。全年单位生产总值能耗下降4.5%，化学需氧量和二氧化硫排放总量分别下降1.78%和2.64%。以创建林业生态市为载体，积极推进国家、省、市林业重点生态工程建设，城乡绿化一体化成效显著。完成工程造林19万亩，新建和完善农田林网30万亩，全市林木覆盖率达到26%。我市林业局被评为全国林业系统先进集体。

（六）狠抓城市建设和管理，加快推进城镇化进程。在巩固“六城二奖”创建成果的基础上，以第三轮城市总体规划修编为龙头，切实加强城市基础设施和公共服务设施建设，提升了城市功能和集聚水平，促进了城市经济发展，城市综合竞争力不断增强。颁布了《濮阳市国家卫生城市管理办法》，实施了城市精细化管理，深入开展了爱国卫生运动，城市建设和管理正向高品位、高质量、精细化转变。建设路东延、石化路东延、苏北路西延、濮上路濮水河桥改造等重点城建项目建成投用，10条背街小巷改造全部完成，新建了10座公厕、10座垃圾中转站，改造了濮水公园，城市绿化覆盖率达到42.7%，市容市貌有了新变化。全面推进新一轮村镇规划修编工作，5个县城的总体规划修编工作已经完成，全市75个乡（镇）中60个乡（镇）新一轮规划编制已按要求完成，县城和小城镇建设有了新变化，全市城镇化率达到32.1%。

（七）改善民生，大力推进和谐濮阳建设。就业和社会保障取得新成绩。城镇新增就业人员8.9万人，实现再就业2.6万人，消除零就业家庭629户。全市城镇登记失业率3.6%，低于4%的控制目标。企业参保离退休人员养老金发放率100%。通过失业保险金扩大支出范围，支付经济补偿金1423万元、促进就业资金1556万元，惠及7700名失业人员。累计发放城市低保资金5365万元，惠及59342名城市低保对象，实现了动态管理下的应保尽保。按照每人每月不低于30元的补差标准，累计发放农村低保金3382万元，救助农村低保人员99210名。改扩建农村敬老院65所，农村五保户集中供养率达到30%。积极开展流浪乞讨人员救助，共救助人员2951人次。筹措资金332.5万元，帮助灾民重建倒房1043间。对城市困难家庭实施廉租房保障制度，及时发放困难家庭住房保障补贴，惠及住房困难家庭165户。巩固了建设领域工程款和农民工工资清欠成果，有效防止了新的拖欠发生。加大扶贫工作力度，又有3.9万贫困人口解决了温饱。加强文化基础设施建设。实施了农村电影放映“2131”工程，免费为农民放映电影11000场。积极开展送戏下乡活动，巡回演出1000余场。切实改善农村生产生活条件。开工建设了金堤河张庄提排站改扩建工程，金堤河下游内涝问题将得到有效解决。投资1亿元，建设农村公路501公里，全市2977个行政村全部实现村村通水泥（油）路。216个村级组织活动场所建成投用。投资4125.8万元，解决了11.98万人的饮水安全问题。投资3417万元，新建农村户用沼气池2万座。实施了农村电网盲点村改造工程，解决了1.6万户贫困户的用电问题。切实解决城乡群众就医难、上学难问题。社区卫生服务机构达到43家，社区卫生覆盖率达到95%。提前实现了新型农村合作医疗制度基本覆盖全市农村居民的目标，已有241万人参加了新型农村合作医疗，占全市农村人口的81.4%。疾病预防控制工作取得显著成绩，我市被评为全国疾病预防控制工作先进单位。落实农村“两免一补”资金1.32亿元，资助中小学生107万人次。投入3153万元，为农村义务教育阶段学校采购课桌凳19.6万套。投入2550万元，组织实施农村中小学校舍维修改造工程项目176个。投入3124.2万元，完成了1024所农村中小学远程教育工程建设任务。加大城区学校建设力度，市第九中学建成投用。高度重视食品和产品质量安全，深入开展专项整治，实施了食品安全放心工程，严厉打击制售假劣药品行为，维护了广大人民群众的身体健康和生命安全。

深入实施科教兴濮和人才强市战略，组织实施各类科技项目145项，新增民营科技企业30家、省级工程技术研究中心2家、省级科技企业5家、省级高新技术企业7家、省级高新技术产品12种。在与郑州大学、省科学院开展良好合作的基础上，又与河南工业大学、省农科院建立了市校、市院合作关系。基础教育进一步巩固，职业教育、成人教育和高等教育全面发展。普通高考再创佳绩，全市本科上线人数14139人，上线率居全省第一位，有37名考生被清华大学、北京大学录取，连续三年居全省第一。全面加强人口和计划生育工作，全市人口自然增长率5.03‰，低于全省平均水平。全民健身运动蓬勃开展，竞技体育水平不断提高。以“迎奥运”为主题，成功举办了第二届市直职工运动会。在国家和省体育赛事中共获得26枚金牌。加强安全生产工作，各项控制指标均优于省下达的年度控制目标，连续第四年被评为“全省安全生产工作优秀市”。大力整顿和规范市场经济秩序，发展环境不断优化。民族宗教、外事侨务、广播电视、审计、监察、物价、史志、人防、地震、气象、保险、邮政通信、残疾人事业、国防教育、民兵预备役等工作都取得了新的成绩。

（八）狠抓精神文明和民主法制建设，社会大局保持稳定。围绕建设社会主义核心价值体系，加强社会主义荣辱观教育，精神文明创建活动不断深化，社会文明程度进一步提高。民主法制建设不断推进。深入实施《全面推进依法行政实施纲要》，切实加大行政执法监督力度，法制政府建设成效显著。加强法制宣传教育，扎实推进依法治市。自觉接受人大法律监督、工作监督和政协民主监督，认真办理人大代表建议和政协委员提案。支持各民主党派、工商联民主监督、参政议政。双拥共建工作继续推进，军政军民团结进一步加强。认真落实党风廉政建设责任制，深入推进治理商业贿赂专项工作，反腐倡廉取得新成效。加强机关效能建设，政府工作效率进一步提高。依法做好信访工作，及时化解社会矛盾，被评为“全省信访工作先进市”。建立健全应急机制，妥善处置各类突发事件。强化社会治安综合治理，严厉打击各类违法犯罪活动，连续第四年被评为“河南省平安建设先进省辖市”。

各位代表，过去一年成绩的取得，是省委、省政府和市委正确领导的结果，是全市人民共同奋斗的结果。在此，我代表市人民政府向全市广大工人、农民、知识分子、干部、人民解放军指战员、武警官兵、公安干警以及各界人士致以崇高的敬意，表示衷心的感谢！

在肯定成绩的同时，也要清醒地看到我市经济社会发展中面临的深层次矛盾和问题，主要是经济结构不优、农业基础薄弱、服务业发展滞后、自主创新能力较弱，特别是县域经济实力不强，管长远、打基础的重大项目储备不足等等。关系群众切身利益的上学就医、劳动就业、社会保障等还存在不同程度的问题，一些群众的生产生活比较困难；个别地方和部门领导干部存在形式主义和官僚主义等现象。对此，我们一定高度重视，并在今后的工作中认真加以解决。

二、2008年工作的总体要求、奋斗目标和主要任务

2008年是全面贯彻落实党的十七大精神的第一年，经济社会发展面临着新的机遇和挑战。从国际环境看，和平与发展仍然是时代主题。从国内形势看，党的十七大胜利召开，必将激励和推动全国经济社会进入新一轮发展高潮。国家加强和改善宏观调控、大力促进中部地区崛起、扎实推进社会主义新农村建设的战略部署，为科学发展创造了良好的环境。特别是经过近几年的发展，我市经济实力显著增强，各项工作在全省晋位升级，为加快发展提供了坚实基础和条件。同时，也要看到，国际竞争更加激烈，贸易保护主义和投资保护主义加剧；我市经济社会发展中长期积累的突出矛盾和问题还没有根本解决。总之，我们面临的机遇和挑战同在，但总体上看，仍然是机遇大于挑战。我们一定要准确把握国内外经济走势，进一步增强工作的系统性、预见性、主动性，牢牢掌握发展的主动权，推动濮阳又好又快发展。

今年政府工作的总体要求是：全面贯彻党的十七大精神，以邓小平理论和“三个代表”重要思想为指导，深入贯彻落实科学发展观，大力实施以工兴市战略、科教兴濮战略、人才强市战略、开放带动战略、可持续发展战略和品牌城市战略，加快推进工业化、城镇化和农业现代化，着力调整经济结构和转变经济发展方式，着力推进改革开放和自主创新，着力加强节能减排和生态环境保护，更加重视改善民生和促进社会和谐，推动经济社会又好又快发展。

今年经济社会发展的主要预期目标是：生产总值增长13%；地方财政一般预算收入增长15%；全部工业增加值增长16%；全社会固定资产投资增长18%；社会消费品零售总额增长15%；外贸出口增长15%，实际利用外资增长25%；居民消费价格总水平涨幅低于去年；城镇居民人均可支配收入增长8%，农民人均纯收入增长8%；新增城镇就业6万人以上，城镇登记失业率控制在4.5%以内；人口自然增长率控制在6‰以下；单位生产总值能耗降低4.6%，化学需氧量、二氧化硫排放量分别控制在2.62万吨、2.96万吨以内。重点抓好以下十个方面的工作：

（一）坚持新型工业化方向，加快工业经济园区化、规模化、产业化发展步伐。要坚定不移地实施以工兴市战略，按照新型工业化的要求，扩张总量，提升质量，尽快形成以优势产业为主导、以大型企业集团为支撑、以重大产业基地为载体的工业发展新格局。一要大力发展工业集聚区，推动产业集群发展。当前，工业经济的发展与发展空间不适应的矛盾，已成为影响全市工业发展的瓶颈。解决这一问题的根本路子就是大力发展工业园区，推动产业集聚发展。每个县（区）都要按照布局科学、用地集约、产业集聚、功能完善的原则，规划建设工业园区。园区要有规模，留足发展空间。各县（区）要把每年的工业用地指标集中用于园区建设，逐年扩大园区面积。要加快园区基础设施建设和功能区划分，今后凡新上项目一律进园区发展。要对进区项目进行科学规划和论证，按程序完善环评、安评等前置手续。入园项目必须是科技含量高、经济效益好、资源消耗低、符合环保标准、人力资源优势得到充分发挥的大中型项目，严禁高污染、高耗能、低效益的项目在我市落地。市工业园区要加快编制园区总体规划，加快基础设施建设，建立科学的管理体制，奋力打开局面。市高新区、市工业园区要顺应发展的新要求，谋求更大的发展空间，建设成濮阳对外开放的窗口和经济发展的动力基地。二要加快培育大型企业集团，发挥引擎作用。要优化发展环境，全力支持驻濮中央、省属大型企业集团做大做强，成为濮阳经济发展的强大引擎。同时，要在资金、用地、资源等生产要素方面，对地方优势产业中的龙头企业给予强力支持，使其优化资源配置，加快行业整合，形成一批行业地位领先、规模优势明显、产品结构合理、经济效益突出、发展潜力巨大的企业集团，成为濮阳经济发展新的引擎。要抢抓国家加快发展资本市场的机遇，支持濮耐公司完成上市工作，推动蔚林化工、恒润化工、宏业化工、绿能高科尽快上市。要对现有中小企业进行战略重组，扩能改造，促其做大做强。三要加快发展八大优势产业，拉长产业链条。要按照“龙头带动、产业支撑、优化结构、集群发展”的思路和依靠科技进步、劳动者素质提高、管理创新，发展壮大八大优势产业，提升产业竞争力。要支持优势企业兼并重组，拉长产业链条，提高精深加工产品和终端产品比重，使其做大做强。要重点发展现代煤化工、盐化工、精细化工、食品、林纸林板等产业，推动资源性产业由初级加工为主导向精深加工为主导转变。要加快自主创新和高新技术产业发展，支持优势产业关键技术研发，形成一批优势产业自主创新平台。四要坚持项目兴工。集中力量抓好114个投资超千万元的重点工业项目建设，进一步扩张工业经济总量，增加工业经济对全市经济的贡献份额。

（二）把解决“三农”问题作为重中之重，全面推进社会主义新农村建设。一要调整农业结构。进一步提高粮食综合生产能力，确保粮食生产稳定在200万吨左右。要大力发展高效经济作物和特色农产品，建设特色农产品基地，加快形成以水稻、小尖椒、食用菌、花卉、林果等为主的优势产业带。要大力发展现代畜牧业，加快优质畜产品生产及加工基地建设。要发展农业产业化经营，大力扶持13家省级和60家市级重点农业产业化龙头企业做大做强。扶持黄河滩区开发建设，加快黄河滩区农业结构调整。二要加强现代农业基础建设。切实加强耕地特别是基本农田保护，加大中低产田改造力度。要大力推进农田水利基本建设，新增有效灌溉面积2.2万亩，新增旱涝保收田面积2万亩，节水灌溉面积6万亩。完成黄河标准化堤防二期工程建设年度计划任务。推进农业科技进步，加强基层农业技术推广体系建设，建立健全动植物疫病防控体系。大力扶持农民专业合作组织，提高农业组织化程度，引导各类市场主体参与农业产业化经营。健全农村市场流通体系。大力推进农村信息化，完善气象灾害信息预警预报及发布系统，增强防御自然灾害的能力。三要全面推进新农村建设。省委、省政府决定，从今年开始，用3年时间分类推进全省新农村建设，分别明确了三类村庄（人均收入2000元以下的村庄、人均收入2000元至5000元之间的村庄和人均收入5000元以上的村庄）的工作重点和奋斗目标，并且要出台分类推进新农村建设的具体方案。各县（区）和市发改委、市农业局、市新农村建设办公室等单位要深入研究政策措施，结合“学习李连成，建设社会主义新农村”活动，在抓好203个新农村建设示范村和103个扶贫开发重点村的同时，制定全市分类推进新农村建设工作方案。加快农村饮水安全、道路、沼气、电力、文化、体育、卫生等基础设施建设，实施绿色家园行动计划，用3年时间，有效改善环境卫生状况和村容村貌，推动我市社会主义新农村建设再上新台阶。

（三）狠抓基础设施和基础产业重点项目建设，进一步增强经济发展后劲。今年，我市要重点抓好产业和基础设施建设投资超千万元的项目170个，概算总投资234亿元，2008年计划完成投资100亿元。在产业方面：抓好中原大化50万吨甲醇、永煤龙宇化工20万吨甲醇项目建设，争取早日投产；强力推进龙丰纸业25万吨轻量涂布纸、永煤龙宇盐化工、永煤永乐10万吨L—乳酸、清凌乳业10万吨乳制品等项目建设，尽快形成施工高潮；开工建设蔚林化工1.12万吨橡胶助剂扩建，濮耐1万吨氮化硅结合碳化硅砖、1万吨滑板、5000吨连铸三大件，北京林氏集团5万吨聚异戊二烯乳液等项目；做好新广源30万吨烧碱、30万吨聚氯乙烯、濮阳县盐化工等项目前期工作。在基础设施方面：开工建设大广高速豫冀省界至南乐段；全力推进濮范高速公路建设；积极推进工业污水处理厂、城市生活垃圾填埋场、中原油田生活污水处理厂扩建等项目建设；配合做好长治至兖州复线铁路濮阳段、德商高速公路范县段的前期工作；完成仓颉500千伏输变电站扩建工程，扎实推进国电2×60万千瓦发电机组等项目的前期工作。在推进项目建设过程中，要严格执行国家关于加强和规范新开工项目管理的规定，完善项目审批、用地、安评、节能减排等手续，制止盲目建设“两高”和产能过剩行业中的粗加工项目。同时，要充分发挥市场配置资源的作用，强化企业和民间资本的投资主体地位，引导更多的资金进入基础设施和基础产业建设领域。今年确定的170个重点项目，是拉动濮阳当前发展和增强濮阳发展后劲的重要力量，必须举全市之力，强力推进，确保按工期建设竣工投用。

要加快发展现代服务业，提高服务业比重和水平，增强消费对经济的拉动作用。既要发展各种生活性服务业，又要大力发展各类生产性服务业，特别要支持新兴服务业发展，以消费结构升级带动产业结构调整。要加强市场体系建设，加快发展商贸流通业、物流业和建筑业，支持重点物流项目建设。要落实消费政策，改善消费环境，积极扩大城市服务消费，大力启动农村消费，进一步拓宽消费领域。

（四）坚持改革开放，为科学发展提供体制机制保障和强大动力。要按照十七大提出的着力构建充满活力、富有效率、更加开放、有利于科学发展的体制机制的要求，积极稳妥推进各方面体制改革创新和产权制度改革。坚持因企制宜，一厂一策，加快国有企业改革步伐，力争年内全面完成全市国有工业企业改革任务，全面推进国有非工业企业改革。要通过大力引进战略投资者，引领全市经济结构调整和产业升级。要加快建立国有资本经营预算制度，完善国有资产监督管理体制，确保国有资产保值增值。要按照国家和省要求，积极推进行政管理体制、农村公路管养体制、事业单位改革，强力推进市政、公用等建设领域改革，切实做好医药卫生、科技、教育、财税、金融、商贸、价格、投资等各项改革。

要大力实施开放带动主战略，加大招商引资力度，借助外力加快濮阳发展。一要更加重视招大商、引大资。认真总结引进永煤、国电、省投资集团等战略投资者的经验和成功做法，积极与国内外大公司、大财团对接，力争更多的世界500强和国内100强企业落户濮阳，成为濮阳未来产业发展的引擎和龙头。同时，要积极参加第三届中国中部投资贸易博览会、第五届河南投资贸易洽谈会、豫港投资贸易洽谈会等大型商贸文化活动，多渠道引进市外资本。要承办好2008＇河南海外联谊会，实现宣传濮阳、招商引资的目的。二要突出园区招商和产业招商。要学习借鉴天津、威海等经济发达地区的先进经验和做法，高起点搞好园区规划和产业规划，把规划好的产业和项目推向国内外同类产业的地区和企业进行招大商、引大资，实施战略合作，形成大项目落地、产业链拉长、配套产业群集聚，从而扩大我市产业规模、增强发展的后劲。三要加强对签约项目的跟踪问效，提高项目履约率。要完善项目落实机制和责任追究制，重点抓好近年来市里组织的五次大型招商活动签约的146个项目的全程跟踪服务，提高项目履约率，促使项目早日开工建设。

（五）坚持统筹城乡协调发展，不断壮大县域经济实力。一要提升区域中心城市品位，增强其辐射带动作用。要全面贯彻落实《城乡规划法》，严格按照我市第三轮城市总体规划修编、专业规划、详细规划，指导城市建设和管理。今后凡政府投资建设的工程项目一律实行代建制，以确保工程质量，提高投资效益。要加强城市道路、供排水、供热等各种基础设施建设，拉大城市框架，完善城市功能。要加大西部城区建设开发力度，运用市场经济的机制，聚集各方建设力量，高标准设计，高水平建设，打造城市新亮点，展示濮阳新形象。要通过穿靴戴帽洗脸的方式，改造沿街建筑景观，使市容市貌有较大的改观。二要推进市、县老城区和“城中村”改造。要坚持政府引导、统一规划、市场运作、群众自愿的原则，积极吸引社会资金参与老城区和“城中村”改造，盘活存量土地，加快城市建设。要学习省内外成功的做法，突出重点，分类推进，创造性地工作，力争2010年前全面完成老城区和“城中村”改造任务，使城市旧貌换新颜。要积极吸引开发商参与县城改造和建设，完善县城功能，改变县城面貌。要把老城区改造与建设特色街区、大力发展城市服务业结合起来，进一步优化城区产业结构。三要进一步加强城市管理。按照《濮阳市国家卫生城市管理办法》要求，落实目标管理责任制，完善长效管理机制，创新管理理念、体制和手段，突出抓好市容环境、市政设施、园林绿化、公共交通等方面的精细化管理，巩固和发展“六城二奖”创建成果，建设宜居城市。

坚持统筹城乡发展、区域经济协调发展，按照省统一部署，制定全市主体功能区规划，明确主体功能区定位和开发方向，优化经济布局。县域经济是我市经济发展的基石和长期抓手，必须集中力量加快发展，强力突破。各县（区）都要强化责任意识和位次意识，紧紧依托资源优势，选择一至两个特色产业，科学规划，加大政策扶持力度，做大做强产业龙头。要把发展壮大非公有制经济作为发展县域经济的主要抓手，落实政策，营造环境，强力突破。要引导、支持特色产业龙头积极寻找战略合作者，实现规模扩张，拉长产业链条，加快县域经济发展。

（六）坚持做好财税金融工作，为经济社会发展提供财力支撑。只有发展才能使财源滚滚而来，只有不断的发展才能不断开辟新兴财源和后续财源，构筑多元化效益型财源格局。为此，我们要坚定不移的把抓发展作为培植财源的重要手段，促进财政增收。要强化税收征管，严格依法治税，严厉打击偷、逃、抗、骗税行为，做到应收尽收，确保今年财政预算任务完成，增强财政对经济社会的保障能力。要调整和优化支出结构，增加社会保障、卫生、教育等民生方面支出，加大对农业和农村发展、自主创新、能源资源节约、生态环保等重点领域的投入力度，保障经济社会发展薄弱环节和公共服务支出需要。要进一步提高预算编制的科学性和约束性，把部门正常经费足额列入年度部门预算，原则上不再追加预算。对各部门各单位不经市委、市政府同意进行的任何超预算的活动，市财政一律不予考虑费用追加。要严格控制一般性支出增长，坚决制止铺张浪费，提高财政资金使用效益。要加强对支农惠农资金的监管，严禁截留、挪用各项支农资金。各金融机构要以支持濮阳发展为己任，用足用活政策，扩大贷款投放，加大对中小企业的支持力度，促进企业加快发展，实现银企双赢。要抓紧做好市城信社不良资产置换工作，提高其资本充足率，尽快组建城市商业银行。

（七）坚持社会主义先进文化方向，推动文化大发展大繁荣。一要大力发展文化事业。要大力发展公益性文化事业，加快农村广播电视覆盖、文化馆、图书馆和乡镇综合文化站建设。加强重点文物和非物质文化遗产保护、开发和利用。坚持不懈地开展“扫黄打非”斗争。二要加快发展文化产业。要按照市场主导、政府引导、财政补贴的办法推动文化产业基础设施和配套工程建设。要用好文化产业发展专项资金，重点培育龙头文化企业和文化品牌。要整合全市杂技文化资源，运用市场机制，打造一台杂技艺术精品。要大力发展独具特色的地方文化，对清丰孝道、濮阳县舜帝、范县郑板桥等文化现象进行挖掘创新，弘扬优秀传统文化。要促进各类文化产业竞相发展，形成文化品牌和产业优势。要推动文化产业与高科技相结合，加快有线电视网络整合和数字化改造。要促进文化、科技与旅游相融合，培育旅游精品，拓展旅游产业链，做大旅游产业。要完善文化产业项目库，搞好文化产业招商引资，加强文化交流与合作。建立健全文化连锁经营、物流配送、电子商务等现代流通组织形式，完善现代文化市场营销体系。积极引导和扩大文化消费，加强文化市场监管。三要深化文化体制改革。加快由办文化为主向管文化为主转变。继续深化文化馆、报社、电台、电视台等公益性文化事业单位内部人事、分配和社会保障制度改革。加快艺术团体、出版单位、新华书店、文化经营中介机构等经营性文化事业单位转制为企业，妥善解决国有文化资产管理、人员分流安置和社会保障等问题。创新大型文化设施管理理念和经营机制，盘活文化资源。鼓励引导非公有制资本进入文艺表演、文化旅游和书报音像制品分销等文化产业领域。

（八）坚持统筹发展社会事业和改善民生，强力推进和谐濮阳建设。一要大力实施科教兴濮战略和人才强市战略。加强科技创新体系建设，着力做好市级重点实验室、市级以上工程技术研究中心的建设工作。搞好科技成果的引进、示范和推广，加大科技成果转化力度，今年安排科技计划项目100项。切实保护知识产权，扶持具有自主知识产权的技术、标准和品牌，促进自主知识产权成果产业化。建设人才公共服务体系，加强创新型人才和各类紧缺人才的引进、选拔和培养。优先发展教育事业。巩固提高义务教育，促进义务教育均等发展。不断提高高中阶段教育水平，大力发展职业教育，支持高等教育提高教学质量和教学改革工程。积极发展民办教育，鼓励和规范社会力量办学。完善教育资助体系，保障经济困难家庭子女平等接受义务教育。加快教育基础设施建设，新建、扩建一批中小学校舍，着力解决中小学生入学难问题。更加关注并解决残障弱智孩子接受义务教育问题。二要大力发展医疗卫生事业。加快推进医疗卫生体制改革，建立健全覆盖城乡居民的公共卫生服务、医疗服务、医疗保障和药品供应保障体系。完善城乡医疗救助制度，重点加强农村三级卫生服务网络和城市社区卫生服务网络建设，重视重大传染病预防控制和职业病防治工作。扶持中医药事业发展。统一实施县级以上医院药品网上招标采购，实现同种药品全市同价。三要认真做好就业再就业和收入分配工作。认真落实《就业促进法》，建立健全创业服务体系和平等就业制度，做好高校毕业生、退伍转业军人、残疾人就业工作。要认真贯彻《劳动合同法》和《劳动争议仲裁法》，规范和协调劳动关系，依法维护劳动者权益。深化收入分配制度改革，建立职工工资正常增长和支付保障机制，逐步提高最低工资标准。继续做好规范公务员津贴补贴工作。实施职工带薪休假制度。切实解决好拖欠职工工资和农民工工资问题。四要完善社会保障体系。积极推进事业单位养老保险制度改革，探索建立农村养老保险制度。加强社保资金监管，确保基金安全。积极推行政策性农业保险。进一步做好城乡低保工作，实现应保尽保。大力发展以扶老、助残、救孤、济困为重点的社会福利和慈善事业。进一步扩大住房公积金覆盖面，加强资金监管和使用安全。落实政策，加强监管，保持市场物价基本稳定，保障市场供应。完善对低收入群众补助办法，切实保障困难群众基本生活。五要加强社会管理。创新社会管理体制机制，积极发挥各类社会组织的作用。做好第六届村委会换届选举工作。进一步落实安全生产责任制，狠抓各项措施的落实和安全隐患的整治，坚决遏制重特大事故发生。加强信访工作，加强人民群众与政府的联系，有效预防和化解各类社会矛盾。健全社会治安防控体系，依法防范和打击违法犯罪活动，深入开展平安创建活动。加强市场监管，确保产品质量和食品药品安全。坚持计划生育基本国策，稳定低生育水平，优化人口结构，提高人口素质。与此同时，坚持统筹兼顾，继续做好外事侨务、广播电视、统计、保险、地震、史志、邮政通信等工作。

今年，市政府要办好与全市人民群众生产生活息息相关的十二大实事：（1）加大对农民的补贴力度。多方筹措资金，继续做好对种粮农民直接补贴、小麦良种补贴、棉花良种补贴、农村劳动力转移培训补贴、测土配方施肥补贴、农业生产资料综合直补和农机具购置补贴，确保补贴资金落实到位。落实好奶牛和生猪生产扶持政策。积极组织好农民购买家电直接补贴的试点工作。（2）切实改善农村村容村貌。指导各县（区）搞好村镇建设规划，从环境卫生综合整治入手，加强农村基础设施建设。筹措资金7000万元，改造县乡公路80公里、通村公路120公里。筹措资金4000万元，解决9万农村居民的饮水安全问题。筹措资金4000万元，新增农村户用沼气2万户，积极推进农村改厨、改圈、改厕。大力推进“万村千乡市场工程”，再建515个农村便民超市。实施农村小康环保行动计划，重点监督指导人均收入超5000元的行政村开展生态文明村建设。启动林业生态市建设村镇绿化工程。（3）强力推进农业基础设施建设。筹措资金4000万元，开工建设濮阳县、清丰县、南乐县大型商品粮基地，使我市优质小麦扩大20万亩。筹措资金4000万元，新增优质水稻面积6万亩。筹措资金8600万元，完成张庄提排站改扩建工程。加大投入力度，扶持41个畜牧养殖小区。继续实施防沙治沙工程，发展林下经济10万亩。（4）大力发展城乡文体事业。推进农村电影放映工程，使60%的行政村实现一村一月放映一场公益性电影。开展“百部流动舞台千场演出送农民”活动。实施430个行政村文化信息资源共享工程。扩建改造5个乡镇综合文化站。开展迎奥运系列全民健身活动，完成220个村农民体育健身试点工程建设。支持社区文化、广场文化等群众性文化活动。（5）进一步提高义务教育保障水平。为农村义务教育阶段学生全部免费提供教科书，并逐步建立教科书循环使用机制。提高农村中小学生人均公用经费标准，确保小学达到230元、初中达到375元。提高农村经济困难家庭寄宿生生活费补助标准，小学、初中由每名学生每天1元分别提高到2元和3元。从2008年秋季全面免除城市义务教育阶段学生学杂费。继续实施农村中小学图书教学仪器设备充实工程。再改造11所农村初中校舍，改善10所城区薄弱学校的办学条件。积极解决农民工子女入学和城区大班额问题。（6）继续改善群众医疗卫生条件。对23个前期投入不足的乡镇卫生院配备基本医疗设备。建成9所乡镇卫生院病房楼，全面完成乡镇中心卫生院改建任务。按照“三级甲等”中医院标准，改扩建市中医院。在全市农村全面实行新型农村合作医疗制度，参合标准由每人50元提高到100元。认真实施国家扩大免疫规划，将甲肝、流脑等15种可以通过接种疫苗有效预防的传染病纳入国家免疫规划范围。加强免疫规划冷链系统建设。全面开展城镇居民基本医疗保险。高风险企业农民工基本参加工伤保险。筹措资金500万元，免费为5000例白内障患者实行复明手术。（7）扎实推进城市低收入家庭住房保障工作。开工建设经济适用房15万平方米，竣工5万平方米，为城市低收入家庭提供700套经济适用房。新建、收购廉租住房1万平方米，为城市低保住房困难户提供200套廉租房房源。加大廉租住房租赁补贴发放力度，惠及住房困难家庭2000户以上。（8）认真做好就业再就业工作。确保全年城镇新增就业 6万人以上，实现下岗失业人员再就业 1.5 万人，动态消除“零就业家庭”。继续实施“阳光工程”、扶贫培训“雨露计划”和农村劳动力技能就业计划，完成5万农民工务工技能培训，新增6万农村富余劳动力转移就业。（9）加强社会保障和对城乡困难群众的救助。继续提高企业退休职工基本养老金水平，人均月增加100元以上。将城市低保对象人均月补差标准由70元提高到95元。将农村低保标准由不低于30元提高到不低于40元。筹措资金800万元，新建、改建和扩建农村敬老院，争取农村五保对象集中供养率达到40%。将集中供养五保对象的生活标准由每人每年1200元提高到1400元，分散供养五保对象的生活标准由每人每年1000元提高到1100元。加大扶贫开发力度，完成40个贫困村的整村脱贫任务，使全市3.5万农村贫困人口脱贫和解决温饱。（10）努力保障食品药品安全。强化食品、药品产品质量监管，实施食品放心工程，开展食品安全专项整治，及时查处食品、药品质量安全违法违规案件，使全市城市、农村食品抽检合格率分别提高1个和2个百分点。鼓励药品连锁和集中配送向农村发展，筹措资金建设农村药品监督网和供应网。（11）积极推进污染防治。开工建设城市西部工业区污水处理厂。加快城市生活垃圾处理场建设，推进市污水处理厂改造升级，加快县级污水处理厂配套管网建设和运营管理，使污水处理厂、垃圾处理场全部实现达标排放、稳定运行。加强饮用水源地安全建设，完成饮用水源地保护规划编制。（12）提升城市建设品位。完成建设路油田供应处段等城市道路建设。对马颊河、濮水河进行综合治理，使河水变清，提高沿岸景观水平。改善提升人民路街景使之成为示范街区。强化城市精细化管理，建设宜居城市。

（九）节约资源、保护环境，强力推进生态文明建设。一要抓好节能减排工作。要严格实行节能减排问责制和“一票否决”制，打好节能减排攻坚战。要认真执行重点产品能耗限额标准，抓好落后生产工艺、设备和产品的淘汰工作。要支持和鼓励重点行业、重点企业应用先进的节能新技术、新设备，不断提高能源利用效率，积极推广生产过程中余热、废气和废液回收利用等节能环保关键技术。加强对新建项目的节能环保准入审查。要推广多层标准厂房，提高土地集约利用水平。要继续开展土地“三项整治”，加强对闲置和低效土地的利用，对已关闭黏土砖瓦窑厂年内完成土地复垦工作。积极推广新型墙材，县以上城市全面禁止使用实心黏土砖。推进农村、建筑行业、交通运输、政府机构等领域的节能工作。继续抓好重点区域和行业的环境综合整治，搞好马颊河、濮水河、濮清南引黄工程等沿线的环境保护。加强燃煤电厂除尘脱硫设施建设和运行监管。加快建立完善城乡垃圾收集清运处理系统。控制农村面源污染。推动污水、垃圾处理等产业化。二要大力发展循环经济。编制实施循环经济发展方案，用好政策，全面推进各行业各领域的循环经济发展。争取中原大化成为全省循环经济试点。加快国电中水工程建设进度，确保上半年实现中水再利用。支持蔚林化工和宏业化工利用国债资金实施清洁生产项目。支持再生资源循环利用企业发展，大力推进废旧资源再生利用。三要扎实推进林业生态市建设。全面实施林业生态市建设规划，完成林业生态市建设各项目标任务，使我市生态环境承载能力显著提高，生态文明建设力争走在全省前列。

（十）坚持依法治市，进一步加强精神文明建设。深入开展“ 五五”普法教育，提高全民法律素质。认真落实《各级人民代表大会常务委员会监督法》，自觉接受人大及其常委会的法律监督、工作监督和人民政协的民主监督，严格执行人大及其常委会的决议、决定，支持人大代表依法行使职权，认真办理人大代表议案、建议和政协委员提案。广泛听取各民主党派、工商联和无党派人士的意见和建议，充分发挥工会、共青团、妇联等人民团体的桥梁纽带作用。全面贯彻党的民族宗教工作基本方针，发挥宗教界人士和信教群众在促进经济社会发展中的积极作用，进一步加强民族团结。加强法律援助，依法保障公民特别是妇女、未成年人和残疾人的合法权益。

以社会主义核心价值体系为重点，加强理想信念教育和思想道德建设。大力弘扬爱国主义、集体主义、社会主义思想。以培育文明风尚和增强诚信意识为重点，加强社会公德、职业道德、家庭美德、个人品德建设，引导人们自觉履行法定义务、社会责任、家庭责任。搞好廉政文化建设。加强和改进思想政治工作，注重人文关怀和心理疏导。重视青少年思想道德教育工作，为青少年健康成长创造良好社会环境。加强网络文化建设和管理。积极开展全民阅读活动。广泛开展科教、文体、法律、卫生进社区和文化、科技、卫生“三下乡”活动，加强广场文化、社区文化、企业文化、校园文化等精神文明建设。深入开展文明城市创建活动，确保“全国创建文明城市工作先进城市”荣誉称号，争创“全国文明城市”。

深入开展国防教育，支持驻濮解放军和武警部队建设，不断提高预备役部队、民兵和人民防空建设质量。认真落实军人优待抚恤和退伍安置政策，做好军队转业干部、退役军人安置工作。积极开展双拥共建活动，巩固和发展军政军民团结。

三﹑加强政府自身建设

要适应改革开放和现代化建设的新形势，着力推动政府自身改革和建设，努力做到为人民服务、对人民负责、受人民监督、让人民满意，加快建设服务型政府，为濮阳发展提供有力保障。

（一）依法行政，科学决策。各级政府、所有公务员特别是各级领导干部，都要严格按照法定权限和程序履行职责，将依法行政作为政府工作的基本准则，严格依法办事，努力建设法制政府。要完善行政管理决策机制，健全对涉及全局的重大事项决策协商和协调机制，强化决策咨询，重视调查研究，推进行政决策科学化、民主化。依法规范行政行为，用制度管权、管事、管人，加强对领导干部、关键岗位的监督，健全质询、问责、经济责任审计、引咎辞职等制度，强化行政执法监督，严格实行行政执法责任制和执法过错责任追究制。搞好行政复议。要深入落实行政许可法，进一步减少和规范行政审批事项。要大力整顿市场经济秩序，全面落实促进经济发展的各项政策，创造诚信法治、公平竞争的市场环境，依法保护投资者的合法权益。

（二）执政为民，提高公共服务水平。要继续推进政企分开、政资分开、政事分开、政府与市场中介组织分开，减少对微观经济运行的干预。要健全政府职责体系，在加强经济调节和市场监管的同时，更加注重履行社会管理和公共服务职能，完善公共服务体系，优化公共资源配置，着力改善民生和加强社会建设，维护社会公平与正义。要认真落实国务院《政府信息公开条例》，大力推进政务公开，保障人民群众的知情权、参与权、表达权和监督权。积极推进学校、医院等单位和电力、通信等行业服务公开制度。要加强和完善各级行政服务中心建设，提高办事效率，切实为社会、企业和人民群众提供优质便捷服务。按照国家、省统一安排部署，搞好经济普查、污染源普查和土地调查，为科学决策提供参考。完善应急预案体系和管理体制，有效应对自然灾害、事故灾难、公共卫生和社会安全突发事件，提高危机管理和抗风险能力。

（三）求真务实，转变工作作风。坚持求真务实、真抓实干的工作作风，时刻保持积极进取、昂扬向上的精神状态，强化大局意识、长远意识、忧患意识，增强责任感、使命感，认真负责地做好各项工作。进一步转变思想作风、领导作风和工作作风，多做打基础、管长远的工作，多解决影响改革发展稳定的突出问题，多办为人民群众排忧解难的实事，切实提高公信力、执行力。大力精简会议、文件，改进会风、文风，力戒形式主义和官僚主义。牢记“两个务必”，厉行节约、勤俭办事，严格财政支出管理，严格控制修建楼堂馆所，严格公费出国出境管理，严格规范公务接待行为，坚决反对铺张浪费和奢靡之风，建设节约型机关。要加强对公务员的教育、管理和监督，进一步提高公务员管理的法制化、规范化水平，努力建设一支政治坚定、业务精通、清正廉洁、作风优良的公务员队伍。加强审计、监察工作，加大源头治理力度，实行标本兼治，进一步抓好政府系统党风廉政建设责任制的落实。大力推行效能问责制，实施“治庸计划”，着力解决行政不作为、乱作为、不会作为问题，提高执政能力，优化发展环境，努力加快濮阳发展步伐。

各位代表！做好今年的政府工作，意义重大。让我们紧紧团结在以胡锦涛同志为总书记的党中央周围，认真贯彻落实党的十七大精神，高举中国特色社会主义伟大旗帜，深入贯彻落实科学发展观，团结拼搏，开拓进取，为实现全面建设小康社会目标、建设富裕文明和谐新濮阳而努力奋斗！
